# Validity and reliability of hallux valgus angle measurement on smartphone digital photographs

**DOI:** 10.1186/s13047-023-00670-8

**Published:** 2023-10-16

**Authors:** Albert Cakar, Ozkan Kose, Firat Dogruoz, Huseyin Selcuk, Tolga Kirtis, Omer Faruk Egerci

**Affiliations:** 1 Istanbul Training and Research Hospital, Department of Orthopedics and Traumatology, Istanbul, Turkey; 2 Antalya Training and Research Hospital, Department of Orthopedics and Traumatology, Varlık mah, Kazım Karabekir cd Address Muratpasa, Antalya, 07100 Turkey

**Keywords:** Hallux valgus, Hallux valgus angle, Margo medialis pedis, Photograph, Diagnosis, Smartphone

## Abstract

**Background:**

This prospective study aimed to test the reliability and validity of hallux valgus angle (HVA) measurement on smartphone digital photographs compared with the standard radiographic evaluation.

**Methods:**

Twenty Seven female patients (45 feet) with forefoot deformity were evaluated with weight-bearing anteroposterior foot radiographs and smartphone photographs. Radiographic hallux valgus angle (rHVA) was measured on digital radiographs. Two different photographic HVA measurement methods were used. In the first, the longitudinal axes of the first metatarsal and proximal phalanx were determined, and the angle between these axes was measured (pHVA), similar to the radiographic method. In the other method, the angle of the margo medialis pedis was measured on the photograph (pMMP). Two independent observers performed all measurements twice on two different occasions. Reliability analysis was performed using the interclass correlation coefficient. Agreement between the measurements was tested using Bland-Altman analysis.

**Results:**

The repeated rHVA, pHVA and pMMP measurements showed excellent intra and inter-observer reliability, with ICC values above 0.900. The mean rHVA, pHVA, and pMMP were statistically similar (p:0.929, 27.03°±8.7°, 27.11°±8.8° and 26.5°±9.0° respectively). The mean difference between the rHVA and pHVA was − 0.07°±5.1° (range, --9.67 to 9.56°), and the mean difference between the rHVA and pMMP was 0.53°±4.4° (range, -9.76° to 8.22°). There was a strong positive correlation between both photographic methods and radiographic measurements (rho = 0.809, *p* = 0.001 and rho = 0.872, *p* = 0.001). In the Bland Altman plot, the upper and lower LOAs (95%CI) ranged from − 10.11° to 9.93° for rHVA and pHVA, and from − 8.26° to 9.33° for rHVA and pMMP. Linear regression analysis showed a proportional bias for pHVA but not for the pMMP (p:0.010 versus p:0.633, respectively). The range of the mean difference (prediction interval) between the pMMP and rHVA was 17.59° and 20° for pHVA and rHVA. Simple linear regression showed that the rHVA was predicted by the following equation: rHVA = 4.73 + 0.84 × pMMP (r2 = 0.761, *p* < 0.001).

**Conclusions:**

Although measuring HVA through smartphone photographs is reliable, it is not a valid prediction method.

**Level of evidence:**

Level II, diagnostic assessment.

## Introduction

Accurate measurement of the hallux valgus angle (HVA) is crucial for assessing the severity of the deformity and guiding treatment decisions. Radiographic evaluation has traditionally been the gold standard for quantifying HVA [1]. However, the advancement of digital photography and image analysis techniques has opened new possibilities for the non-invasive assessment of hallux valgus (HV) using photographic measurements. In addition, these technologies have been integrated into the smartphone and have found widespread use [2]. It has been suggested that this method offers convenience and precise measurements while avoiding exposure to ionizing radiation associated with radiographs.

To date, few studies have investigated the reliability and validity of photographic measurements in assessing the HVA [3–5]. These studies have shown high correlation and acceptable validity between photographic measurements and weight-bearing radiographs, demonstrating the potential of this method for evaluating HV deformity. However, a comprehensive comparison between photographic and radiographic HVA measurements is needed further to validate the reliability and accuracy of the photographic assessment.

This study aimed to conduct a comparative analysis of photographic HVA measurements with radiographic measurements. By evaluating the agreement and correlation between these two methods, we aim to assess the feasibility and accuracy of photographic evaluation as a potential alternative to radiographic assessment in clinical practice. The findings from this study may have implications for improving diagnostic accuracy, monitoring treatment outcomes, and reducing radiation exposure in patients with HV. Professionals who do not have direct access to radiographic facilities, such as podiatrists and physiotherapists, can also utilize these measurements. Furthermore, photographic assessments might also be used for large-scale epidemiologic studies.

## Materials and methods

### Patients and study design

This prospective study was conducted in the authors’ institution between May and August 2023. Before the initiation of the study, IRB approved the study protocol, and the study followed the declaration of Helsinki and its later amendments (Approval date:10.02.2023, Issue:31). All participants provided written informed consent. Adult patients who were admitted to the outpatient clinic with complaints of forefoot deformity and possible diagnosis of HV were included in the study. Patients who declined to participate and patients with a previous history of forefoot surgical procedures or fractures and congenital deformities were excluded from the study.

### Sample size calculation

We used the Bland-Altman plot approach to determine the required sample size for our study, comparing the photographic HVA and radiographic HVA measurement methods. Based on a previous study that reported a mean difference of -5.3° and 95% limits of agreement of -15.9° to 5.3°, we estimated the standard deviation between the methods to be 2.06° [5]. However, since we also had access to a regression equation that predicted the values from the pHVA values, we incorporated this information into our calculation of the required sample size. Using the formula for the standard deviation of the residuals, we estimated the standard deviation of the differences to be 1.62°, which we used in our sample size calculation. Assuming a maximum acceptable difference of 2°and a desired power of 80% with a significance level of 0.05, we calculated that a sample size of at least 31 feet would be needed for our study.

### Radiographic and photographic measurements

Anteroposterior weight-bearing radiographs were taken with the x-ray beam inclined at 20° from vertical in the sagittal plane at a distance of 100 cm, directed vertically to the cassette in the coronal plane, and centered in the middle of the third metatarsal. All radiographs were taken with the same digital X-ray machine (Arcoma Intuition X-ray System, Comp-Ray, Phoenix, AZ, USA). HVA was measured using the software program MicroDicom DICOM Viewer (ver. 2022.3, MicroDicom Ltd, Bulgaria) on the digital workstation. The measurement methodology followed the recommendations provided by the ad hoc committee of the American Orthopaedic Foot and Ankle Society [6].

**Fig. 1:**
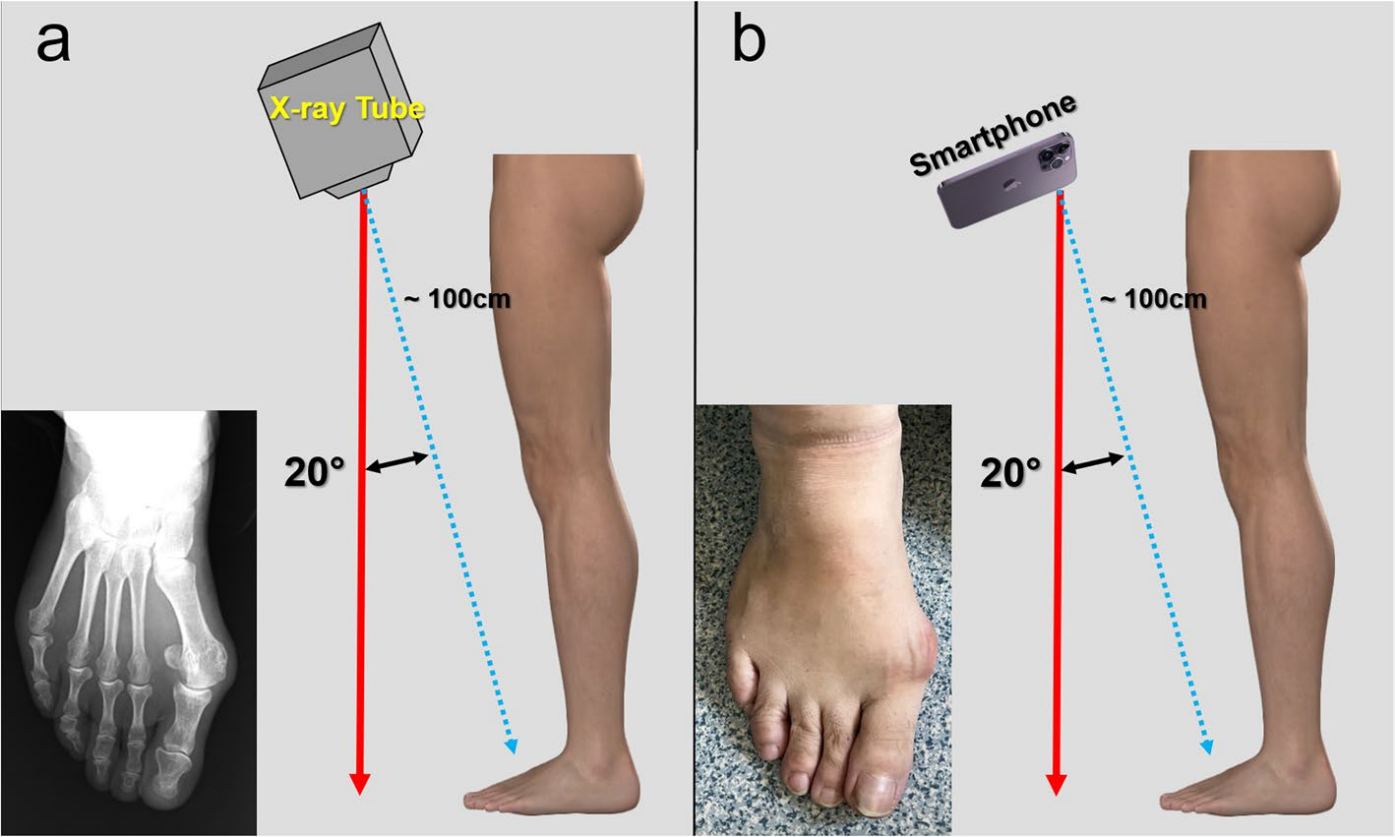
**a** Radiographic imaging technique. **b** Photographic imaging technique

**Fig. 2:**
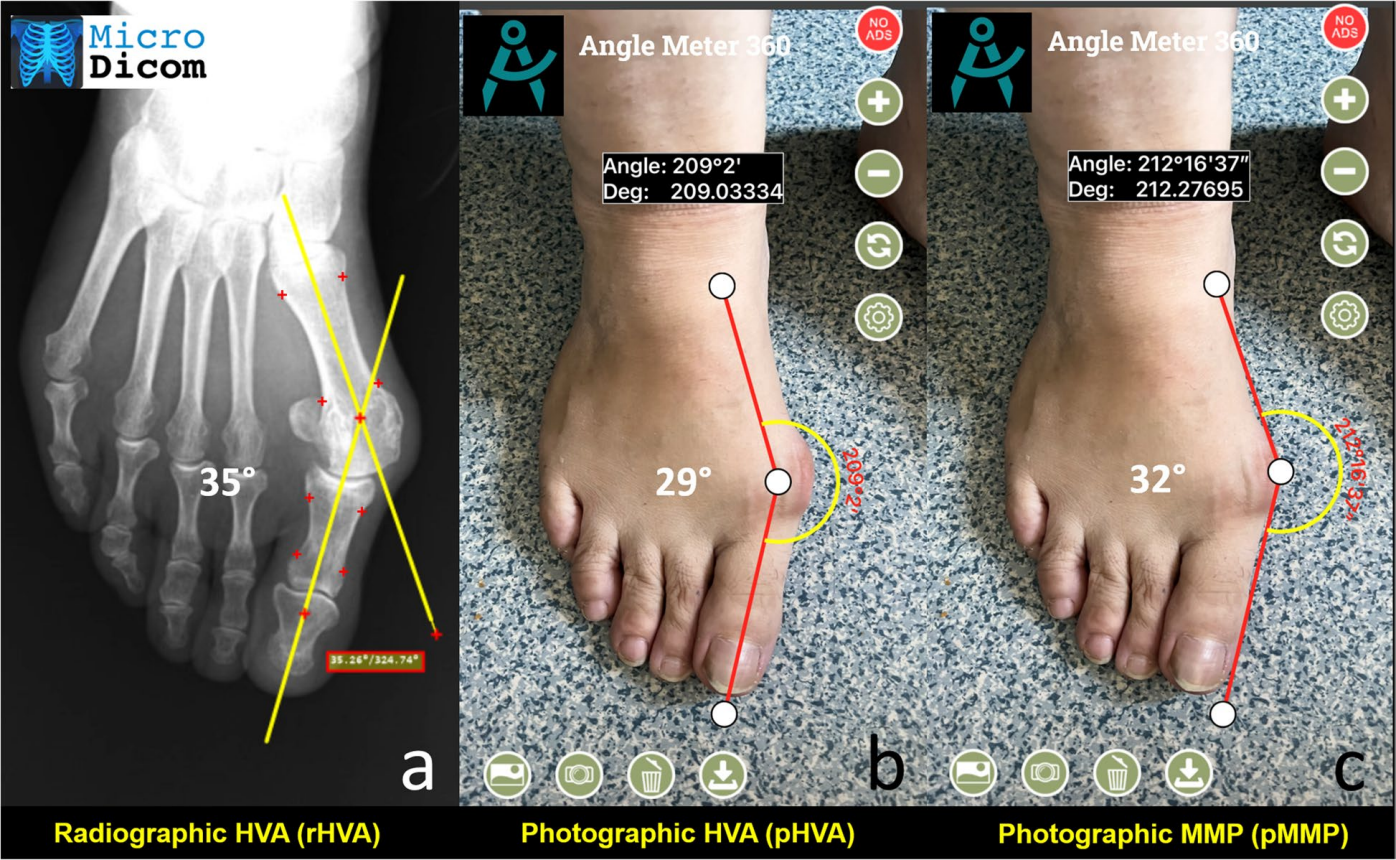
**a** Radiographic HVA measurement. **b** Photographic HVA (pHVA) measurement. **c** Photographic margo medialis pedis (pMMP) measurement

Foot photographs were taken while the patient was standing, similar to the weight-bearing radiographs. An iPhone 14 ProMax was used to take photographs. The camera was held parallel to the dorsum of the foot around 20° inclined to the ground, and the foot was fit into the screen (Fig. 1). The same author took all foot photographs. All smartphone measurements were performed using an Apple iPhone (Apple, Cupertino, CA, USA) running the Anglemeter software (WG Healthcare UK, Herts, UK), which was downloaded from the Apple iTunes store as a free application [7]. The photographs contained no identifying markers, and observers were blinded to the patient’s radiologic measurements. Given the insights from previous studies, two distinct measurements were conducted on the photographs [3–5]. The first measurement was executed employing a method analogous to the radiologic HVA measurement, subsequently termed photographic HVA (pHVA). Relying on the foot’s topographic anatomy, the longitudinal axis of the first metatarsal was delineated by identifying the base and head of the first metatarsal. Subsequently, the longitudinal axis of the proximal phalanx was delineated. The angle formed between these two lines was measured and documented. In the secondary measurement methodology, the medial border of the foot and the medial border of the big toe were utilized as references; thus, this angle was designated as the photographic margo medialis pedis angle (pMMP). The distal line originated at the metatarsal head, extending along the edge of the proximal phalanx, while the proximal line was drawn from the metatarsal head to the navicular process, ensuring a meticulous representation of anatomical alignments (Fig. 2).

### Testing the reliability of the measurements

Two consultant orthopedic surgeons took part in the study. All radiographs and photographs were measured twice on two different occasions at least three weeks apart. Observers were blinded to their own and other observers’ ratings. The photographs and radiographs were anonymized and shuffled on each occasion.

### Statistical analysis

Descriptive statistics were presented with mean, standard deviation, and range. Normality was assessed using the Kolmogorov–Smirnov test. Intra-observer and interobserver reliability was calculated using the interclass correlation coefficient (ICC). ICCs of 0.90–1.00, 0.75–0.90, 0.50–0.75, and > 0.50 were interpreted as excellent, good, moderate, and poor, respectively [8]. ANOVA and student t-tests were used to compare the independent measurements. Pearson correlation was used to analyze the correlation between the variables. Simple regression and Bland-Altman plot analysis were used to determine the level of agreement (LOA) between different measurement methods. A *p*-value less than 0.05 was set as statistically significant.

## Results

There were 27 female patients (18 bilateral cases, total 45 feet) with a mean age of 46.6 ± 10.4 years (range, 24–68). The rHVA, pHVA and pMMP measurements showed excellent intra and inter-observer reliability, with ICC values above 0.900 (Table 1). Overall, the reliability of the measurements was within acceptable limits, and the mean of four measurements for each variable was used for the rest of the analysis.

**Table 1:** Intra and inter-observer reliability of the repeated measurements by the observers

**Variables**	**Intra-observer Reliability**	**ICC (95% CI)**	**Interpretation**
**rHVA**	***Observer A t***_***1***_ *** vs.t***_***2***_	0.974 (0.953-0.986)	Excellent
	***Observer B t***_***1***_ *** vs.t***_***2***_	0.931 (0.878-0.961)	Excellent
**pHVA**	***Observer A t***_***1***_ *** vs.t***_***2***_	0.908 (0.832-0.949)	Excellent
	***Observer B t***_***1***_ *** vs.t***_***2***_	0.915 (0.850-0.952)	Excellent
**pMMP**	***Observer A t***_***1***_ *** vs.t***_***2***_	0.937 (0.888-0.965)	Excellent
	***Observer B t***_***1***_ *** vs.t***_***2***_	0.953 (0.916-0.974)	Excellent
	**Inter-observer Reliability**		
	***Observer A t***_***1***_ *** vs. B t***_***2***_	0.935 (0.885-0.964)	Excellent
**rHVA**	***Observer A t***_***2***_ *** vs.t***_***2***_	0.971 (0.948-0.984)	Excellent
	***Observer A t***_***1***_ *** vs.t***_***1***_	0.907 (0.830-0.949)	Excellent
**pHVA**	***Observer A t***_***2***_ *** vs. B t***_***2***_	0.923 (0.860-0.958)	Excellent
	***Observer A t***_***1***_ *** vs.t***_***1***_	0.961 (0.931-0.979)	Excellent
**pMMP**	***Observer A t***_***2***_ *** vs. B t***_***2***_	0.938 (0.890-0.965)	Excellent
	***Observer A t***_***1***_ *** vs.t***_***2***_	0.974 (0.953-0.986)	Excellent

*Abbreviations rHVA* radiographic hallux valgus angle, *pHVA* photographic hallux valgus angle, *pMMP* photographic margo medialis pedis, *ICC* Interclass correlation coefficient, *t*_*1*_ Time 1, *t*_*2*_ Time 2, *CI* Confidence interval

**Fig. 3:**
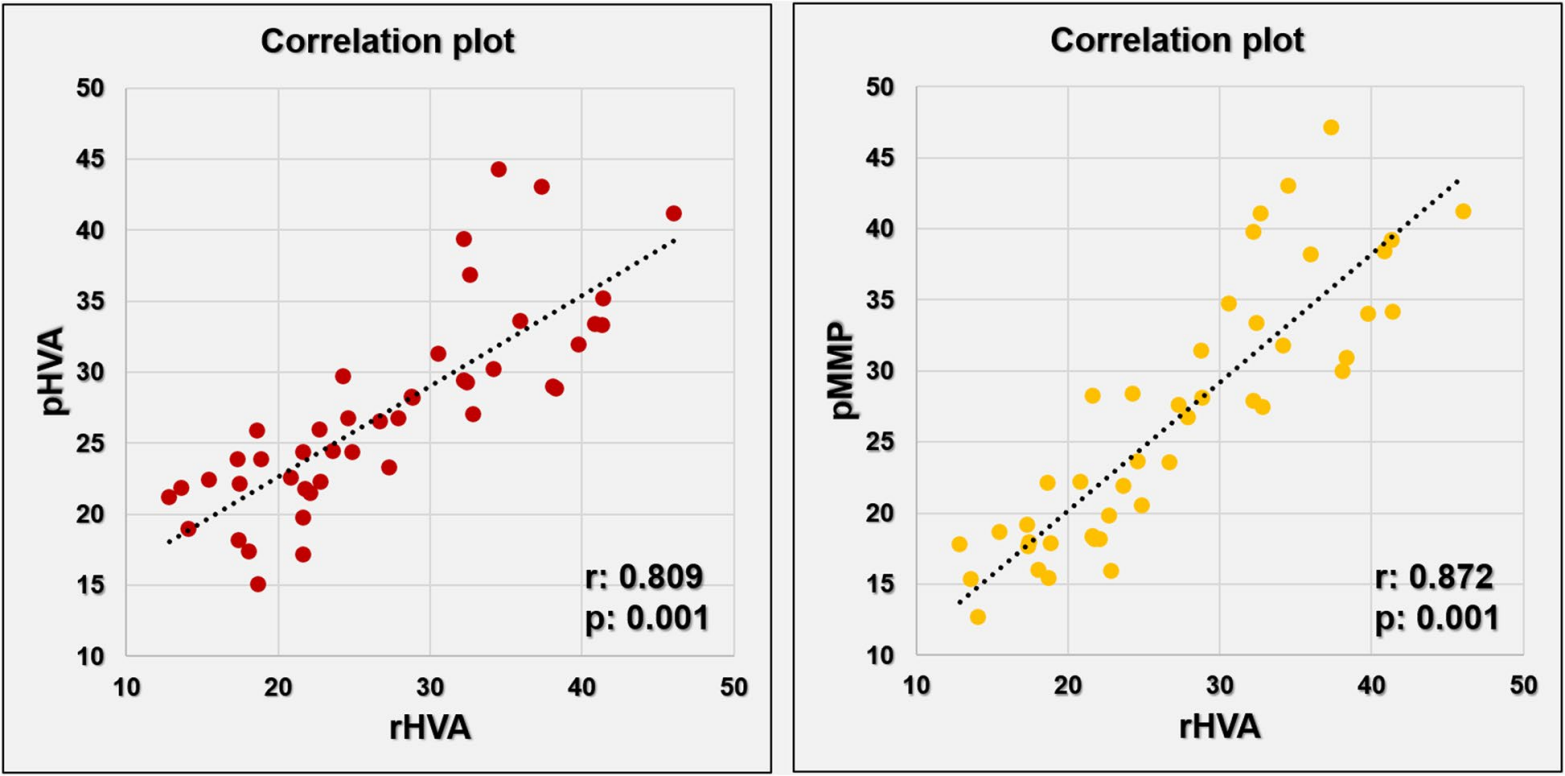
Correlation plots between rHVA and pHVA, and rHVA and pMMP

The mean rHVA, pHVA, and pMMP were statistically similar (p:0.929, 27.03°±8.7°, 27.11°±8.8° and 26.5°±9.0° respectively). The mean difference between the rHVA and pHVA was − 0.07°±5.1° (range, --9.67 to 9.56°), and the mean difference between the rHVA and pMMP was 0.53°±4.4° (range, -9.76° to 8.22°). There were no significant differences between the mean bias and the test value zero in one sample t-test for both photographic methods (Table 2). Photographic methods and radiographic measurements showed a strong positive correlation (Fig. 3).

**Table 2:** The mean measurements and their comparison

**Variables**	**Mean°±SD**	**Min-Max (°)**	***p*** **-value**
**rHVA**	27.03°±8.7°	12.87–46.10	0.929^*^
**pHVA**	27.11°±8.8°	15.06–44.22	
**pMMP**	26.50°±9.0°	12.64–47.12	
**Δ rHVA-pHVA**	-0.07°±5.1°	-9.67-9.56	0.917^**^
**Δ rHVA-pMMP**	0.53°±4.4°	-9.76-8.22	0.430^**^
**|Δ rHVA-pHVA|**	4.14°±2.9°	0.05–9.67	
**|Δ rHVA-pMMP|**	3.71°±2.5°	0.21–9.76	

*Abbreviations, rHVA *radiographic hallux valgus angle, *pHVA *photographic hallux valgus angle,* pMMP *photographic margo medialis pedis,* Δ *Difference

^*^ANOVA

^**^One-sample t-test, test value:0

**Fig. 4:**
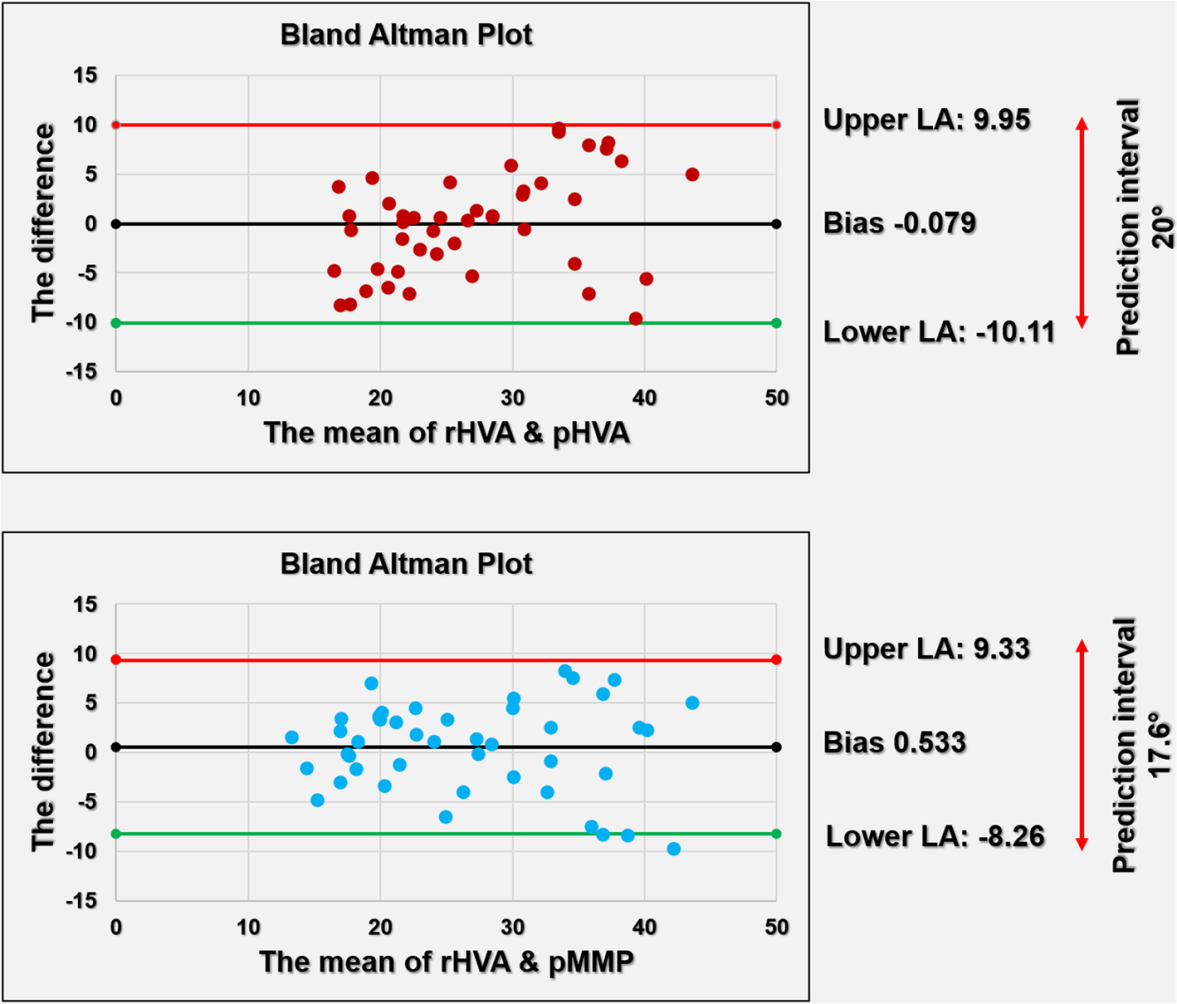
Bland Altman plots showing the LOA between rHVA and photographic methods

In the Bland Altman plot, the upper and lower LOAs (95%CI) ranged from − 10.11° to 9.93° for rHVA and pHVA, and from − 8.26° to 9.33° for rHVA and pMMP (Fig. 4). Linear regression analysis showed a proportional bias for pHVA but not for the pMMP (p:0.010 versus p:0.633, respectively). The range of the mean difference (prediction interval) between the pMMP and rHVA was 17.59° and 20° for pHVA and rHVA. Simple linear regression showed that the rHVA was predicted by the following equation: rHVA = 4.73 + 0.84 × pMMP (*r*^2^ = 0.761, *p* < 0.001).

## Discussion

In this study, we have tested whether the measurement of HVA on smartphone photographs can be used instead of the gold standard radiographic HVA measurement. The findings of this study suggest that the measurement of photographic HVA using pMMP and pHVA methods through a smartphone was repeatable and reproducible, with excellent ICC values. However, both pMMP and pHVA measurements do not appear to be valid prediction methods since the prediction interval reached up to 20°. Previous studies have shown that up to 6.5 degrees of variation might occur between experienced observers, even with standardized radiological techniques [8, 9]. Such a large measurement error is unacceptable, resulting in completely wrong decision-making for treating HV deformity. It is important to note that while smartphone-based measurements have shown promise, they should not replace radiographic evaluation entirely. Radiographs remain indispensable for accurate and comprehensive assessment, especially in cases that require surgical intervention or when detailed measurements are essential for treatment planning.

The disagreement between radiographic and photographic measurements might be related to several reasons. Although we obtained photographic and radiographic shots with similar techniques (equal distance and inclination), shooting errors may have caused wide differences. In our study, the camera was held by the researcher at around 20 degrees inclined to the ground. This approach might reflect real-world clinical practice but varies significantly from methods in studies such as by Nix et al., where the camera was maintained flat against the X-ray tube to mimic the exact angle of the radiographic images [3]. Changes in camera angle may have caused shape distortions in the photographs, resulting in differences between photographic and radiographic measurements. Secondly, the soft tissue landmarks and the underlying bone are probably subjected to individual anatomical variations, such as the foot size, the presence of other foot deformities, the magnitude of the deformity, pes planus, and soft tissue thickness. Identification of the longitudinal axis of the first metatarsal necessitates a good knowledge of topographic anatomy. For these reasons, achieving a truly high-consistency HVA measurement through photographs is challenging. This approach was not employed in the current study; however, the joint lines and the long axes of the first metatarsal and proximal phalanx might have been more accurately determined through palpation and demarcation with a surgical pencil. It is plausible that this methodology could have yielded more accurate results compared to the identification of anatomical landmarks solely through photographic examination, particularly in the measurement of pHVA. Nonetheless, it is generally straightforward to discern anatomical landmarks during pMMP measurement.

Three previous studies investigated photographic HVA measurements to predict the radiographic HVA and tested the reliability and agreement between these techniques (Table 3) [3–5]. Two different photographic measurement methods were used in these studies, namely pHVA and pMMP. Nix et al. found that digital pHVA measurements were reliable and had acceptable validity compared to weight-bearing rHVA measurements. They reported narrower LOAs between the two methods compared to the current study [3]. Hayatoshi et al. compared pMMP measurement with rHVA and reported a statistically significant correlation, suggesting that the smartphone method is a reliable and valid alternative to conventional radiography [4]. However, we think that the statistical analysis conducted in their study is incomplete. High correlation and statistical similarity of average values may give erroneous results in reporting the agreement of the methods. In our study, there was a high correlation, and there was no difference between the means of the methods. However, the LOA was unacceptably wide. Yamaguchi et al. compared the HVA measured using self-photograph and radiography and reported a systematic 5° error [6]. pMMP angle underestimated the rHVA. Similar to our results, they found a wide LAO between pMMP and rHVA (-16.5° to 6.5°). They suggested the use of photographic measurements for screening purposes. The current study also found an unacceptable discrepancy between the radiographic and photographic methods, but photographs might be used for a rough estimation of the severity of the HV deformity.

**Table 3:** Previously published studies that compare radiographic HVA measurements with non-invasive methods

**Author**	**Year**	**# of feet**	**Gold standard**	**Alternative Methods**	**Conclusion**
**Nix et al. **[3]	2012	76	rHVA	pHVA	pHVA is reliable and valid.
**Hayatoshi et al. **[4]	2018	37	rHVA	pMMP	pMMP is equivalent to rHVA.
**Yamaguchi et al. **[5]	2019	100	rHVA	pMMP	pMMP underestimated the rHVA with a 5° systematic error.
**Current Study**	2023	40	rHVA	pHVA & pMMP	pMMP and pHVA are not valid methods to predict rHVA.

*Abbreviations*, *rHVA *radiographic hallux valgus angle, *pHVA *photographic hallux valgus angle,* pMMP *photographic margo medialis pedis

Recently, Inoue et al. [10] attempted to estimate the radiographic parameters for HV, namely HVA, IMA 1–2, and IMA 4–5, from photography using a deep convolutional neural network (CNN). They produced a CNN model and the estimated HVA with their automatic prediction model without user intervention. There was a substantial agreement between the CNN model and the true radiographic measurements (r^2^ = 0.684, root mean squared error = 7.91). Although this preliminary study failed to demonstrate an excellent agreement, advances in artificial intelligence, deep learning, and the processing of large numbers of data, precision may increase, and estimation within acceptable limits may be possible in the near future.

Besides these studies, other non-invasive techniques have been used to measure the HVA, such as footprint measurements, clinical goniometry, and 3D laser scanning systems. Choung et al. compared clinical goniometric measurements with radiographic HVA measurements and reported that goniometer measurements of the HVA are inaccurate and have unacceptable validity [11]. Janssen et al. studied the agreement between goniometric HVA and computerized plantar pressure measurements against gold standard rHVA. The prediction interval was too wide and unsatisfactory for both methods [12]. Zhou et al. measured the HVA on 3D models obtained with laser scanners. They compared footprint measurements and 3D model measurements against rHVA. They showed that, 3D model measurements were highly correlated with rHVA [13].

This study has several strengths as well as limitations. The sample size was calculated, and sufficient subjects were included in the study to reach adequate statistical power. Two independent observers performed the measurements on two separate occasions which increased the accuracy of the data. Since this was a prospective study, data acquisition was standardized. The inclusion of only female patients might prevent the generalizability of the findings over both genders. Both observers were experienced surgeons, the participation of trainees or inexperienced surgeons would provide real-life situations.

In conclusion, this study failed to show that photographic HVA measurements can be used instead of radiographic measurements in clinical settings. It cannot be considered a valid method as it may result in around 10° larger or smaller values. However, pMMP might be used for screening purposes considering possible errors. In the future, incorporating artificial intelligence and developing new photographic processing technologies might allow precise predictions.

## Data Availability

The datasets used and/or analyzed during the current study are available from the corresponding author upon reasonable request.

## Abbreviations

HV: Hallux valgus
HVA: Hallux valgus angle
rHVA: Radiographic hallux valgus angle
pHVA: Photographic hallux valgus angle
pMMP: Photographic Margo medialis pedis
ICC: Interclass correlation coefficient
CI: Confidence interval
SD: Standard deviation
